# Acetylene hydrochlorination over tin nitrogen based catalysts: effect of nitrogen carbon-dots as nitrogen precursor

**DOI:** 10.3906/kim-2102-67

**Published:** 2021-10-19

**Authors:** Yibo WU, Fuxiang LI, Qingbin LI, Songtian LI, Ganqing ZHAO, Xuerong SUN, Peisong LIU, Guoxv HE, Yongjun HAN, Liping CHENG, Shiying LUO

**Affiliations:** 1 College of Chemistry and Enviromental Engineering, Pingding Shan University, Pingding Shan China; 2 College of Chemistry and Chemical Engineering, Taiyuan University of technology, Taiyuan China

**Keywords:** Trichlorophenylstannane, nitrogen carbon-dots, vinyl chloride, acetylene hydrochlorination

## Abstract

The catalysts comprising the main active compounds of Sn-Nx were synthesized using trichlorophenylstannane ((C_6_H_5_)Cl_3_Sn), nitrogen carbon-dots (NCDs), and activated carbon (AC) as starting materials, and the activity and stability of catalysts was evaluated in the acetylene hydrochlorination. According to the results on the physical and chemical properties of catalysts (TEM, XRD, BET, XPS and TG), it is concluded that NCDs@AC can increase (C_6_H_5_)Cl_3_Sn dispersity, retard the coke deposition of (C_6_H_5_)Cl_3_Sn/AC and lessen the loss of (C_6_H_5_)Cl_3_Sn, thereby further promoting the stability of (C_6_H_5_)Cl_3_Sn/AC. Based on the characterization results of C_2_H_2_-TPD and HCl adsorption experiments, we proposed that the existence of Sn-N_x_ can effectively strengthen the reactants adsorption of catalysts. By combing the FT-IR, C_2_H_2_-TPD and Rideal-Eley mechanism, the catalytic mechanism, in which C_2_H_2_ is firstly adsorbed on (C_6_H_5_)Cl_3_Sn to form (C_6_H_5_)Cl_3_Sn-C_2_H_2_ and then reacted with HCl to produce vinyl chloride, is proposed.

## 1. Introduction 

Organotin compounds (OTCs), as one of organometallic compounds [1], have been widely applied to many areas, encompassing anticancer therapy [2–4], biocides [5], stabilizer [6], and catalysts [7–11]. Currently, scientists have proved that alkyl-organotin not only can be used as stabilizer in polymerization vinyl chloride fabrication (PVC) but also can catalyze acetylene hydrochlorination to manufacture vinyl chloride [6,11,12]. The production of PVC in many regions mainly depends on the carbon-supported HgCl_2 _catalytic acetylene hydrochlorination reaction [13]. However, the prevention and governance of mercury pollution is still a serious problem around the world [14,15]. Therefore, the nonmercuric catalytic acetylene hydrochlorination process for PVC production need to be urgently explored.

Great attentions have been paid to study the tin-based catalysts in the hydrochlorination of acetylene, especially inorganic tin in the past few years. Researchers founded that metal compounds additives, including BiCl_3_ [16], ZnCl_2_ [17], CuCl_2_ [18], CeCl_3_ [19], CoCl_2_ [20] and LiCl [21] can effectively improve the stability of inorganic tin-based catalysts. Basing from the study by Deng reported that the deactivation of SnCl_4_/AC catalysts for acetylene hydrochlorination is due to the high volatility of SnCl_4 _[16]. Gao et al. reported that the CoCl_2_ and BiCl_3 _promoters can effectively lessen the coking formation of SnCl_4_/AC in acetylene hydrochlorination and improve the stability of SnCl_4_/AC catalysts [17]. Although SnCl_2_/AC can rival the catalytic activity of Hg-based catalysts, the key scientific and technological issue of SnCl_2_/AC catalysts is to improve its stability [16]. Later on, Guo et al. founded that the synergistic effect of SnCl_2_, ZnCl_2_ and Tb_4_O_7_ can strengthen the catalytic performance of SnCl_2_/AC in the hydrochlorination of acetylene [20]. In our previous work, we reported that LiCl-promoted SnCl_2 _catalysing acetylene hydrochlorination with considerable stability, originating from the synergistic effect between Sn and Li [21]. Moreover, organotin catalysts, as another kind of tin-based catalysts in acetylene hydrochlorination, have been studied [11,12,22]. There is no research regarding the catalytic mechanism of organotin as catalysts for acetylene hydrochlorination.

Owing to the specified electronic properties and unique chemical characteristics, a series of nitrogen-doped carbon materials as catalyst carries is grabbing more attention in acetylene hydrochlorination [23-25]. For example, Dai et al. synthesized g-C_3_N_4_/AC as a novel nonmetallic catalyst, which can catalyze acetylene to produce vinyl chloride [23]. Afterward, scientists reported that the coexistence of pyridinic nitrogen and pyrrolic nitrogen in nitrogen-doped carbons could strengthen the adsorption of the reactants [24]. Furthermore, Li et al*.* studied that the enhancement of AuCl_3_/PPy-MWCNT catalytic performance was due to the electron transfer from N atom in PPy to the Au^3+ ^and, thus, improve the hydrogen chloride adsorption [25]. 

In this paper, nitrogen-carbon quantum dots (NCDs) with distincitive physical and chemical properties [26–30] is used as nitrogen sources in the preparation of tin nitrogen based acetylene hydrochlorination catalysts. Specifically, the aims of this work are to study the effect of NCDs on the performance of (C_6_H_5_)Cl_3_Sn-based catalysts for acetylene hydrochlorination and the catalytic mechanism of (C_6_H_5_)Cl_3_Sn/AC in acetylene hydrochlorination.

## 2. Materials and methods

### 2.1. Materials

Coal-based activated carbon (φ=1.5 mm, l=5 mm) was obtained from Shanxi Xinhua Chemical Company. Trichlorophenylstannane (98.0%) was purchased from TCI (Shanghai) Development Co., Ltd. Citric acid (99.5%), urea (99.0%), ammonium hydroxide (25~28%), and ethanol (99.5%) were obtained from Tianjin Kermel Technology Development Co., Ltd. 

### 2.2. Catalyst preparation

#### 2.2.1. Preparation of NCDs

N-doped carbon quantum dots (NCDs) were prepared by citric acid and urea [31,32]. Specifically, citric acid (2.0 g) and urea (2.0 g) were dissolved in distilled water (15 mL). Then, the mixture was transferred to a Teflon coated stainless-steel autoclave and heated at 160 °C for 7 h. Afterward, the obtained NCDs solution was mixed with ethanol and centrifuged at 8000 rpm for 20 min. Finally, the samples were dried overnight at 80 °C to obtain the purified NCDs powders.

#### 2.2.2. Preparation of NCDs@AC

NCDs (1.0 g), AC (9.0 g) and ammonium hydroxide was mixed in deionized water (20 mL), and the mixture was stirred at a room temperature for 30 min. Then, the mixture was transferred to a Teflon coated stainless-steel autoclave and heated at 200 °C for 8 h. The obtained samples was washed by deionized water and dried at 100 °C overnight. The final solid sample was labeled as NCDs@AC. Carbon support was pretreated by the same procedure, and the obtained carbon sample was denoted as AC.

#### 2.2.3. Preparation of (C6H5)Cl3Sn-based catalysts

(C_6_H_5_)Cl_3_Sn/NCDs@AC was synthesized using NCDs@AC as support and (C_6_H_5_)Cl_3_Sn as active compounds, respectively. Specifically, (C_6_H_5_)Cl_3_Sn (1.5 g) was dissolved in the appropriate amount of ethanol, and then this impregnation solution was slowly added to NCDs@AC (8.5 g). The obtained heterogeneous solid was dried at 80 °C to get 15%(C_6_H_5_)Cl_3_Sn/NCDs@AC. The similar procedure was repeated to prepare the (C_6_H_5_)Cl_3_Sn/AC catalysts for comparisons.

### 2.3. Catalyst characterization

Powder X-ray diffraction (XRD) patterns were carried out on Shimadzu XRD-6000 with Cu Ka radiation (0.15418 nm). All samples were taken at range of 10–80°C. Catalysts were degassed at 150 °C for 4 h, before the nitrogen adsorption/desorption isotherms at –196 °C were analyzed using Quantachrome NOVA 2000e. Fourier transform infrared (FT-IR) spectra were recorded by a Biorad Excalibur FTS 3000 equipped with a DTGS detector. Transmission electron microscopy (TEM) was performed on JEM-2100F instruments at an acceleration voltage of 200 kV, used to study the dispersion of Sn species, and it characterized the morphology of Sn-based catalysts . X-ray photoelectron spectroscopy (XPS) was conducted on EscaLab 250Xi instruments using Al Ka X-ray source and analyzed the valence of element on the surface of support. The spectra were analyzed using XPSPEAK software pack and corrected for changing in using C1s binding energy (BE) as the reference at 284.8eV. Acetylene-temperature programmed desorption (C_2_H_2_-TPD) measurements were performed on a FINESORB-3010 chemisorption analyzer. Briefly, the samples (50 mg) were first treated with Ar gas at 200 °C for 1.5 h. After cooling, it was continually flushed with a C_2_H_2_ flowing at a rate of 25 mL·min^–1^ and heated from room temperature to 500 °C at a heating rate of 10 °C·min^–1^. The coke deposition of spent catalysts were determined by thermogravimetric analysis (TG) instruments (NETZSCH STA 449F3) over the temperature from atmosphere temperature to 800 °C at a heating rate of 15 °C·min^–1^ and an air flow rate of 30 mL·min^–1^. HCl adsorption experiments were analyzed by titration method [22].

### 2.4. Catalyst tests

The hydrochlorination of acetylene tested in the fixed-bed micro-reactor (i.d.10 mm). The reaction temperature was regulated using a temperature controller (Yudian Al-808H). When the reactor temperature initially was maintained at 180 °C, the hydrogen chloride firstly fed into the reactor containing 4 mL catalysts to get rid of moistures and air in reaction system for 30 min. The mole ratio of C_2_H_2_/HCl=1.0:1.1 was calibrated by mass flow controller with a given C_2_H_2_-GHSV of 30 h^-1^. Then, the product mixture gas was getting through the medical soda lime to remove the unreacted hydrogen chloride. The final product gas was analyzed by an online gas chromatograph (GC900) using TCD as the detector for gas chatomatograph, which equipped with a packed column (GDX301). 

## 3. Results and discussion

### 3.1. Physicochemical properties of NCDs

**Figure 1:**
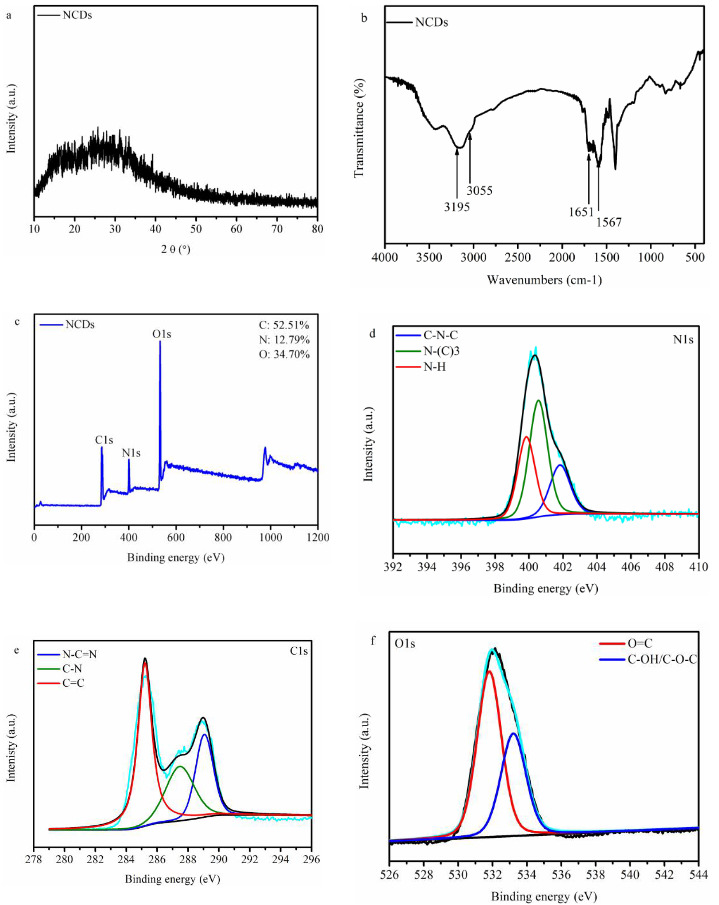
(a) XRD patterns of NCDs, (b) FTIR spectra of NCDs, (c) XPS patterns of NCDs, (d) High resolution XPS-C1s spectra of NCDs, (e) High resolution XPS-N1s spectra of NCDs, (f ) High resolution XPS-O1s spectra of NCDs.

Figure 1a shows that the XRD patterns of NCDs displays a broad peak at 23.8°, suggesting that NCDs is mainly composed of amorphous carbon [33–35]. Figure 1b shows the FT-IR spectra of NCDs, with four characteristic peaks at 3195, 3055, 1651 and 1567 cm^–1^ inferring the bonding formation of -CO-NH- [26]. The composition of NCDs was studied by XPS. The full scan spectra of XPS confirm the existence of C, N and O in NCDs (Figure 1c). The high-resolution N_1s_ is depicted in Figure 1d, three peaks at 399.6 eV, 400.5 eV and 401.7 eV that commonly correspond to C-N-C, N-(C)_3_ and N-H, respectively [27,36] (Figure 1e). In the XPS-C_1s _spectra of NCDs, the deconvoluted three peaks at 284.5 eV, 286.0 eV, and 288.4 eV can be assigned to C=C, C-N, and N-C=N, respectively [26,36]. Moreover, two types oxygen species at 531.8 eV (C=O) and 533.3 eV (C-OH/C-O-C) are observed in the sample (Figure 1f) [36,37]. Based on both FTIR and XPS results, the successful synthesis of NCDs was confirmed.

### 3.2. Catalytic performance

**Figure 2:**
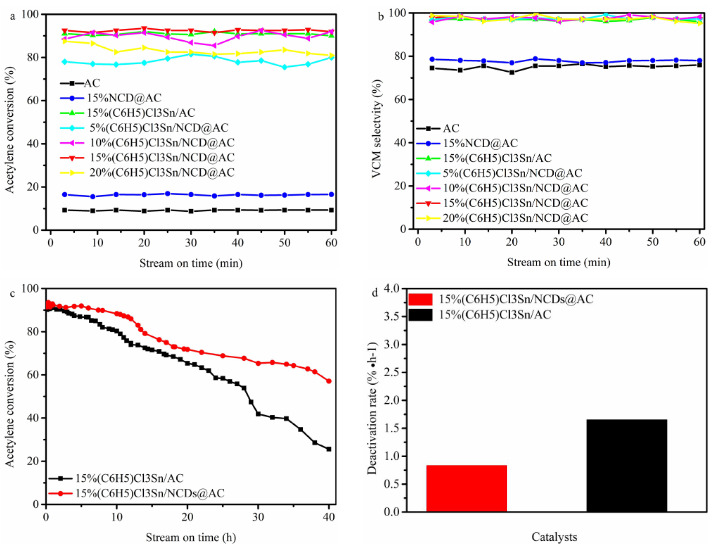
(a) Acetylene conversion and (b) VCM selectivity of different catalysts, (c) Stability of 15%(C6H5)Cl3Sn/NCDs@AC and 15%(C6H5)Cl3Sn/AC, (d) Deactivation rate of 15%(C6H5)Cl3Sn/NCDs@AC and 15%(C6H5)Cl3Sn/AC (Reaction condition: T=180 °C, C2H2-GHSV=30 h-1, VHCl/VC2H2=1.1/1.0).

The effect of NCDs on the performances of (C_6_H_5_)Cl_3_Sn/AC catalysts was evaluated at a temperature of 180 °C with C_2_H_2_-GHSV of 30 h^-1^, and the results is displayed in Figure 2a. The acetylene conversion of 5%(C_6_H_5_)Cl_3_Sn/NCDs@AC, 10%(C_6_H_5_)Cl_3_Sn/NCDs@AC, 15%(C_6_H_5_)Cl_3_Sn/NCDs@AC, and 20%(C_6_H_5_)Cl_3_Sn/ 

NCDs@AC are 78.2%, 88.5%, 92.5%, and 87.3%, respectively, and 15%(C_6_H_5_)Cl_3_Sn/NCDs@AC with highest acetylene conversion (92.5%) was selected for the following studies. The acetylene conversion of 15%(C_6_H_5_)Cl_3_Sn/NCDs@AC and 15%(C_6_H_5_)Cl_3_Sn/AC (90.2%) are similar, which prove that NCDs exhibits a little effect on the catalytic activity of (C_6_H_5_)Cl_3_Sn/AC. Comparing with 15%(C_6_H_5_)Cl_3_Sn/AC (90.2%), only 8.9% C_2_H_2_ has been transformed to vinyl chloride over AC and NCDs@AC features 17.9% acetylene conversion, suggesting that (C_6_H_5_)Cl_3_Sn can catalyze the acetylene hydrochlorination to generate vinyl chloride and NCDs doped-AC can enhance the catalytic performance of AC. At the same time, the VCM selectivity of five catalysts decrease in the following order: 15%(C_6_H_5_)Cl_3_Sn/NCDs@AC (99.2%)> 15%(C_6_H_5_)Cl_3_Sn/AC(98.5%)> NCDs@AC (78.8%)> AC (76.5%) (Figure 2b). Experiments show that the higher VCM selectivity of catalysts is mainly attributed to the (C_6_H_5_)Cl_3_Sn. 

As shown in Figure 2c, 15%(C_6_H_5_)Cl_3_Sn/NCDs@AC features stable catalytic performance at first 12 h of the reaction. After 40 h since the reaction started, the acetylene conversion of 15%(C_6_H_5_)Cl_3_Sn/NCDs@AC gradually decreases from 92.5% to 59.2%. However, 15%(C_6_H_5_)Cl_3_Sn/AC reaches the acetylene conversion of 90.2% and then reduced by 66.1% 40 h after reaction. Furthermore, it is concluded that NCDs additives can prolong the lifetime of (C_6_H_5_)Cl_3_Sn/AC catalysts for acetylene hydrochlorination. Figure 2d shows that the deactivation rate of 15%(C_6_H_5_)Cl_3_Sn/AC (1.65 %·h^–1^) is higher than that of 15%(C_6_H_5_)Cl_3_Sn/NCDs@AC (0.82 %·h^-1^), indirectly proving the above-mentioned conclusion.

### 3.3. Physical properties of catalysts

**Figure 3:**
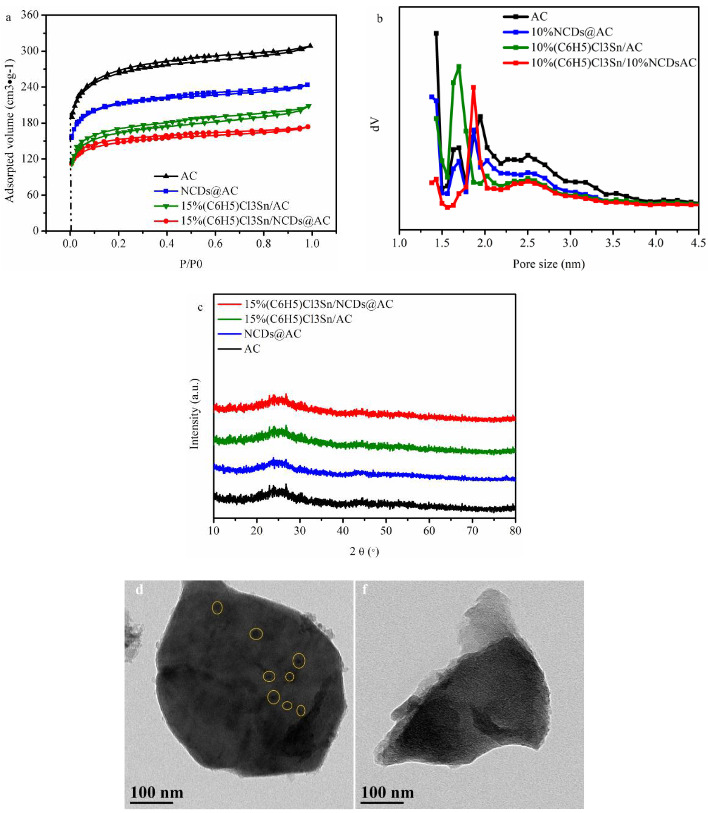
(a) Nitrogen adsorption/desorption isotherm of different catalysts; (b) Pore size distribution of different catalysts; (c) XRD pattern of different catalysts; TEM images of (d) 15%(C6H5)Cl3Sn/AC; (e) 15%(C6H5)Cl3Sn/NCDs@AC

The specific surface area, pore volume, and pore size distribution of catalysts were analyzed by the nitrogen adsorption/desorption experiments. According to IUPAC classification (Figure 3a), all catalysts display the type-I langmuir isotherms and type H_4_ loop, which suggests the coexistence of micro- and mesorpores in samples. This result is in compliance with the pore size distribution curves (Figure 3b). As listed in Table 1, the specific surface area of NCDs@AC, 15%(C_6_H_5_)Cl_3_Sn/AC and 15%(C_6_H_5_)Cl_3_Sn/NCDs@AC is 798 cm^3^·g^-1^, 712 cm^3^·g^-1^ and 631 cm^3^·g^-1^, respectively, which are all lower than that of AC (983 cm^3^·g^-1^), indicating that additives successfully loaded into carbon support. As can be seen in Figure 3c, the two obvious diffraction peaks at 26.4 and 44.4 ° correspond to the (002) and (101) crystal planes of AC (PDF#41-1487), respectively [38]. However, there are no other discernible peaks in 15%(C_6_H_5_)Cl_3_Sn/NCDs@AC, inferring that (C_6_H_5_)Cl_3_Sn homogeneously dispersed on the NCDs@AC surface [39]. We analyzed the 15%(C_6_H_5_)Cl_3_Sn/NCDs@AC and 15%(C_6_H_5_)Cl_3_Sn/AC through TEM images (Figure 3d and Figure 3e). As depicted in Figure 3d, 15%(C_6_H_5_)Cl_3_Sn/AC has some black particles, which represent that (C_6_H_5_)Cl_3_Sn dispersed on the carbon surface. In stark contrast, this phenomenon does not exist in 15%(C_6_H_5_)Cl_3_Sn/NCDs@AC surface. This result suggests that NCDs@AC carrier can promote the better dispersion of (C_6_H_5_)Cl_3_Sn. 

**Table 1:** Texture parameters of catalysts and AC.

Samples	BETtotal	BETmeso	BETmicro	Vtotal	D
	(cm2·g–1)	(cm2 ·g–1)	(cm2·g–1)	(cm3·g–1)	(nm)
AC	983	129	854	0.48	1.90
NCDs@AC	798	89	709	0.41	1.92
15%(C6H5)Cl3Sn/AC	712	106	606	0.38	2.00
15%(C6H5)Cl3Sn/NCDs@AC	631	103	528	0.30	2.20

### 3.4. Chemical properties of catalysts

**Figure 4:**
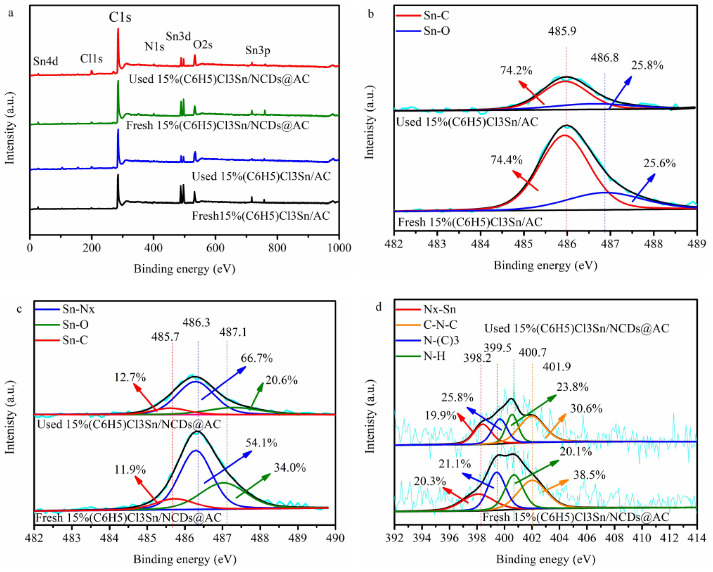
(a) XPS pattern of different catalysts, (b) High resolution XPS spectra of Sn3d5/2 in fresh- and used 15%(C6H5)Cl3Sn/AC, (c) High resolution XPS spectra of Sn3d5/2 in fresh- and used 15%(C6H5)Cl3Sn/NCDs@AC, (d) High resolution XPS spectra of N1s in fresh- and used 15%(C6H5)Cl3Sn/NCDs@AC.

The surface element composition of catalysts and the chemical effect of NCDs on (C_6_H_5_)Cl_3_Sn were investigated by XPS techniques. To be specific, Figure 4a and Table 2 prove that the element of Sn, N, C, Cl and O exist in four (C_6_H_5_)Cl_3_Sn-based catalysts. 

**Table 2:** The relative contents of different elements in catalysts.

Sample	Elements (wt%)				
	Sn	N	C	O	Cl
Fresh 15%(C6H5)Cl3Sn/AC	5.65	0.44	77.84	12.03	4.04
Used 15%(C6H5)Cl3Sn/AC	1.17	0.58	79.72	11.48	7.05
Fresh15%(C6H5)Cl3Sn/NCDs@AC	5.69	2.36	78.01	10.75	3.19
Used15%(C6H5)Cl3Sn/NCDs@AC	2.92	2.04	79.95	11.02	4.07

#### 3.4.1. Sn3d5/2

As shown in Figure 4b, the high resolution Sn3_d5/2_ spectrum of 15%(C_6_H_5_)Cl_3_Sn/AC reaches two peaks at 485.5 eV and 486.9 eV, which are related to the presence of Sn-C and Sn-O, respectively [40–43]. However, the high resolution Sn3_d5/2_ spectrum in the case of 15%(C_6_H_5_)Cl_3_Sn/NCDs@AC (Figure 4c) can be deconvoluted into three individual peaks corresponding to Sn-O (486.9 eV), Sn-C (485.5 eV) and Sn-N_x _(486.0~486.8 eV) [42-45], confirming that the presence of Sn-N_x_ in catalysts is due to the interaction between (C_6_H_5_)Cl_3_Sn and NCDs .

 Because a number of oxygen-containing functional groups on the AC and NCDs surface are able to react with NH_3_ under 200 °C [46], fresh-15%(C_6_H_5_)Cl_3_Sn/NCDs@AC (10.75 wt.%) surface has the lower oxygen element content than that of fresh-15%(C_6_H_5_)Cl_3_Sn/AC (12.03 wt.%) (Table 2). However, in the catalytic acetylene hydrochlorination reaction, the reason of Sn-O in (C_6_H_5_)Cl_3_Sn-based catalysts reacting with HCl may be to generate SnCl_4,_ which easily sublimes at 180 °C, resulting in the loss of Sn species (Table 3). As listed in Table 2, the Sn content in (C_6_H_5_)Cl_3_Sn/AC decreased from 5.65 to 1.17 wt.% after 40 h of reaction. Interestingly, the only Sn amount of 2.92 wt.% was leached from 15%(C_6_H_5_)Cl_3_Sn/NCDs@AC. Additionally, the Sn-N_x_ content in the case of 15%(C_6_H_5_)Cl_3_Sn/NCDs@AC is reduced by 1.09 wt.% after 40 h (Table 4). The above analysis indicated that Sn-N_x _stabilizes the loss of Sn species during the reaction (Figure 2c).

**Table 3:** The relative contents of Sn-O in catalysts.

Sample	Total Sn-O content (wt%)		Loss (wt %)
	Fresh	Used	△Sn-O
15%(C6H5)Cl3Sn/AC	1.45	0.30	1.15
15%(C6H5)Cl3Sn/NCDs@AC	1.93	0.60	1.33

**Table 4:** The relative contents of Sn-Nx in catalysts.

Sample	Total Sn-Nx content(wt%)		Loss (wt %)
	Fresh	Used	△Sn-Nx
15%(C6H5)Cl3Sn/AC	--	--	--
15%(C6H5)Cl3Sn/NCDs@AC	3.07	1.98	1.09

#### 3.4.2. N1s

It can be seen in Figure 4d that there are four fitted peaks (C-N-C (399.6 eV), N-(C)_3_ (400.5 eV), N-H (401.7 eV) and N_x_-Sn (397.7 eV)), in the N1s spectra of 15%(C_6_H_5_)Cl_3_Sn/NCDs@AC [26,38,45], which represents that NCDs successfully dispersed on AC surface and also prove the existence of Sn-N_x_.

**Figure 5:**
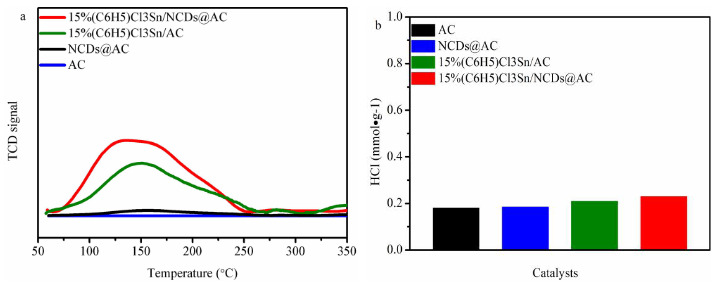
(a) C2H2-TPD of catalysts, (b) HCl adsorption of catalysts.

The effect of NCDs on the reactants adsorption of (C_6_H_5_)Cl_3_Sn-based catalysts was investigated by C_2_H_2_-TPD and hydrogen chloride adsorption/desorption experiments, respectively. The acetylene adsorption amounts of four catalysts are as follows: 15%(C_6_H_5_)Cl_3_Sn/NCDs@AC> 15%(C_6_H_5_)Cl_3_Sn/AC> NCDs@AC> AC (Figure 5a). Particularly, 15%(C_6_H_5_)Cl_3_Sn/AC shows the stronger acetylene adsorption ability than that of AC. This implies that (C_6_H_5_)Cl_3_Sn displays a key role in acetylene adsorption. As illustrated in Figure 5b, 15%(C_6_H_5_)Cl_3_Sn/NCDs@AC, 15%(C_6_H_5_)Cl_3_Sn/AC, NCDs@AC, and AC feature the hydrogen chloride adsorption amount of 0.23 mmol·g^-1^, 0.21 mmol·g^-1^, 0.19 mmol·g^-1^ and 0.18 mmol·g^-1^, respectively (Table 5,6,7,8). Compared to bare AC, the higher content of Pyridine N in NCDs@AC plays a key role on the hydrogen chloride adsorption [47,48]. Furthermore, the hydrogen chloride adsorption of 15%(C_6_H_5_)Cl_3_Sn/NCDs@AC is higher than that of 15%(C_6_H_5_)Cl_3_Sn/AC. It is indicated that NCDs additives can promote the hydrogen chloride adsorption of (C_6_H_5_)Cl_3_Sn/NCDs@AC, as result of the coexistence of Sn-N_x _and Pyridine N (Figure 5b and Figure 4). 

**Table 5:** The different parameter of 15%(C6H5)Cl3Sn/NCDs@AC.

Numbers	Na2CO3(mL)	Adsorption volume(mL)	Catalysts(g)	HCl adsorption capacity(mmol g–1)
1	10530	250	3.51	0.24
2	10033	250	3.49	0.23
3	9652	250	3..51	0.22
4	10063	250	3.50	0.23

### 3.5. Deactivation reason

The specific surface area of 15%(C_6_H_5_)Cl_3_Sn/AC and 15%(C_6_H_5_)Cl_3_Sn/10%NCDs@AC is reduced by 447 cm^2^ g^-1^ and 328 cm^2^ g^-1 ^after 40 h of reaction, respectively (Table 9) indicates indirectly that coke deposition is one of deactivation reason of (C_6_H_5_)Cl_3_Sn/AC in the hydrochlorination of acetylene [49]. Additionally, all the number of tin species in different catalysts decreased promptly in Table 1, which shows that another deactivation occurs from the loss of tin species. 

**Table 9:** Specific surface area of fresh- and used catalysts.

Sample	SBET(cm2g–1)		S△BET(cm2g–1)
	fresh	used	
15%(C6H5)Cl3Sn/AC	712	265	447
15%(C6H5)Cl3Sn/NCDS@AC	631	303	328

### 3.6. Catalysis mechanism

**Figure 6:**
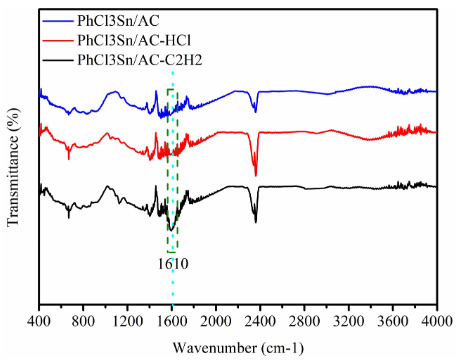
FTIR spectra of different (C6H5)Cl3Sn/AC

The C_2_H_2_-TPD, FT-IR, HCl adsorption/desorption experiments and Rideal–Eley mechanism [50,51] were used to investigate the reaction mechanism of (C_6_H_5_)Cl_3_Sn/AC in acetylene hydrochlorination. In addition, (C_6_H_5_)Cl_3_Sn/AC was separately pretreated with HCl, C_2_H_2_ and N_2 _at 180 °C for 1 h. Later on, (C_6_H_5_)Cl_3_Sn/AC-HCl, (C_6_H_5_)Cl_3_Sn/AC-C_2_H_2_, (C_6_H_5_)Cl_3_Sn/AC-N_2_ were characterized by FT-IR techniques. Both SnCl_4_ and HCl as electron-acceptor do not react with each other [52], and (C_6_H_5_)Cl_3_Sn is more inclined to adsorb acetylene rather than HCl (Figure 5a, b). The above-mentioned two points infer that (C_6_H_5_)Cl_3_Sn prefers to interact with C_2_H_2_ in the catalytic acetylene hydrochlorination process. However, only one characteristic adsorption bands at ~1610 cm^-1^ is observed in (C_6_H_5_)Cl_3_Sn/AC-C_2_H_2_, suggesting that the existence of -C=C- consequently infers the interaction between gaseous C_2_H_2_ and (C_6_H_5_)Cl_3_Sn (Figure 6) [53,54]. This result suggests that the (C_6_H_5_)Cl_3_Sn does interact with C_2_H_2_, and (C_6_H_5_)Cl_3_Sn/AC-C_2_H_2_ is transition state of (C_6_H_5_)Cl_3_Sn in catalysis acetylene hydrochlorination reaction and then adsorbs HCl to generate vinyl chloride. Based on the analysis of previous studies, the reactant adsorption ability of catalysts displays a vital in acetylene hydrochlorination, but strong C_2_H_2_ adsorption may result in deactivation of the catalysts [55–57]. Accordingly, it is proposed that coke deposition is main deactivation reason for (C_6_H_5_)Cl_3_Sn-based catalysts in the hydrochlorination of acetylene. It can be seen from Figure 4b, the binding energy of the Sn-C (Sn3_d5/2_) in (C_6_H_5_)Cl_3_Sn/NCDs@AC is centered at 485.7 eV, but (C_6_H_5_)Cl_3_Sn/AC is located at 485.9 eV. This negative shift is due to Sn-N_x _(Figure 4c). Significantly, this results promote the hydrogen chloride adsorption of (C_6_H_5_)Cl_3_Sn/NCDs@AC and, therefore, improve the catalytic performance of (C_6_H_5_)Cl_3_Sn-based catalysts in the acetylene hydrochlorination reaction.

## 4. Conclusion

In conclusion, (C_6_H_5_)Cl_3_Sn/AC can catalyze the acetylene hydrochlorination process. The intermolecular force between (C_6_H_5_)Cl_3_Sn and NCDs induces the formation of Sn-N_x , _which can promote the (C_6_H_5_)Cl_3_Sn dispersion, reduce the (C_6_H_5_)Cl_3_Sn loss and lessen coke deposition, leading to the longer lifetime of (C_6_H_5_)Cl_3_Sn/AC. According to the Rideal–Eley mechanism and experiments results, we proposed that the (C_6_H_5_)Cl_3_Sn/AC-C_2_H_2_ indicates a transition state of (C_6_H_5_)Cl_3_Sn in catalysis of acetylene hydrochlorination reaction and then adsorbs HCl to generate vinyl chloride. Thus, it is showed that the main deactivation reason of (C_6_H_5_)Cl_3_Sn/AC is coke deposition during the acetylene hydrochlorination. This work provides a novel application of (C_6_H_5_)Cl_3_Sn and NCDs for further studies on the organotin-based catalysts for acetylene hydrochlorination. 

## References

[ref1] Ingham RK Rosenberg SD Gilman H. Organotin Compounds 1960 Chemical Reviews 60 459 539

[ref2] Gielen M. 1996 Tin-based antitumour drugs Coordination Chemistry Reviews 151 41 51

[ref3] Yang P Guo M. 1999 Interactions of organometallic anticancer agents with nucleotides and DNA Coordination Chemistry Reviews 185 189 211

[ref4] Alama A Tasso B Novelli F 2009 Organometallic compounds in oncology: implications of novel organotins as antitumor agents Drug Discovery Today 14 500 508 19429510 10.1016/j.drudis.2009.02.002

[ref5] MA Champ 2000 A review of organotin regulatory strategies, pending actions, related costs and benefits Science of the Total Environment 258 21 71 10.1016/s0048-9697(00)00506-4 11007277

[ref6] Ayrey G Hsu SY Poller RC 1984 The use of organotin compounds in the thermal stabilization of poly(vinyl chloride). VI. an assessment of the relative importance of HCl-scavenging, exchange, and addition reactions Journal of Polymer Science Part A-Polymer Chemistry 22 2871 2886

[ref7] Song J Zhang B Wu T Yang G Han B 2011 Organotin-oxomolybdate coordination polymer as catalyst for synthesis of unsymmetrical organic carbonates Green Chemistry 13 922 927

[ref8] da Silva MA Santos ASS dos Santos TV Meneghetti MR Meneghetti SMP 2017 Organotin(iv) compounds with high catalytic activities and selectivities in the glycerolysis of triacylglycerides Catalysis Science & Technology 7 5750 5757

[ref9] Iwasaki F Maki T Onomura O Nakashima W Matsumura Y. 2000 ChemInform Abstract: chemo-and stereoselective monobenzoylation of 1,2-diols catalyzed by organotin compounds Journal of Organic Chemistry 65 996 1002 10.1021/jo991394j 10814046

[ref10] Khattak ZAK Younus HA Ahmad N Yu B Ullah H 2018 Mono-and dinuclear organotin (IV) complexes for solvent free cycloaddition of CO2 to epoxides at ambient pressure Journal of CO2 Utilization 28 313 318

[ref11] Wu YB Li BW Li FX Xue JW Lv ZP 2018 Synthesis and characteristics of organotin-based catalysts for acetylene hydrochlorination Canadian Journal of Chemistry 96 447 452

[ref12] Wu YB Li FX Xue JW Lv ZP 2020 Effect of various g-C3N4 precursors on the catalytic performance of alkylorganotin-based catalysts in acetylene hydrochlorination Turkish Journal of Chemistry 44 393 408 33488165 10.3906/kim-1909-64 PMC7671198

[ref13] Schobert H 2013 Production of acetylene and acetylene-based chemicals from coal Chemical Reviews 114 1743 1760 24256089 10.1021/cr400276u

[ref14] Gustin MS Amos HM Huang J Matthieu BM Keith H 2015 Measuring and modeling mercury in the atmosphere: a critical review Atmospheric Chemistry and Physics 15 5697 5713

[ref15] Harris HH Pickering IJ George GN 2013 The chemical form of mercury in fish Science 301 1203 1203 10.1126/science.1085941 12947190

[ref16] Deng GC Wu BX Li TS Liu GD Wang LF 1994 Preparation of solid phase non mercury catalyst for synthesis of vinyl chloride by acetylene method Polyvinyl chloride 6 5 9

[ref17] Gao SL Sun X Lv ZL Qin YC Zhang XT 2016 The application of Sn-Bi-Co@AC catalysts for acetylene hydrochlorination Journal of Petrochemical Universities 29 1 5

[ref18] Zhag L Jiang H Wang H Dong SW Ding LW 2013 Preparation and application of non-mercury catalysts for acetylene hydrochlorination Journal of Petrochemical Universities 26 6 11

[ref19] Xiong Q Wu GW Ling S Xiong Z Hu ZP 2007 Preparation and optimization of mercury-free catalyst for synthesis of vinyl chloride from acetylene Modern Chemical Industry 37 66 69

[ref20] Guo YY Liu Y Hu RS Gao GJ Sun HJ 2014 Preparation and optimization of SnCl2-ZnCl2/C mercury-free catalyst for acetylene hydrochlorination Chinese Journal of Applied Chemistry 31 624 626

[ref21] Wu YB Li FX Lv ZP Xue JW 2019 Carbon-supported binary Li-Sn catalyst for acetylene hydrochlorination Journal of Saudi Chemical Society 19 002 002

[ref22] Wu YB Li FX Xue JW Lv ZP 2019 Sn-imidazolates supported on boron and nitrogen-doped activated carbon as novel catalysts for acetylene hydrochlorination Chemical Engineering Communications 207 1203 1215

[ref23] Li X Wang Y Kang L Zhu M Dai B. 2014 A novel, non-metallic graphitic carbon nitride catalyst for acetylene hydrochlorination Journal of Catalysts 311 288 294

[ref24] Lin R Kaiser SK Hauert R Ramírez JP 2018 Descriptors for high-performance nitrogen-doped carbon catalysts in acetylene hydrochlorination ACS Catalysis 8 1114 1121

[ref25] Li X Zhu M Dai B. 2013 AuCl3 on polypyrrole-modified carbon nanotubes as acetylene hydrochlorination catalysts Applied Catalysis, B: Environmental 142 234 240

[ref26] Zhang R 2014 Nitrogen-doped carbon quantum dots: Facile synthesis and application as a“turn-off”fluorescent probe for detection of Hg2+ ions Biosensors & Bioelectronics 55 83 90 24365697 10.1016/j.bios.2013.11.074

[ref27] Yang Z Xu M Liu Y He F Gao F 2014 Nitrogen-doped, carbon-rich, highly photoluminescent carbon dots from ammonium citrate Nanoscale 6 1890 1895 24362823 10.1039/c3nr05380f

[ref28] Dong Y Pang H Yang HB Guo CX Shao JW 2013 Carbon-based dots Co-doped with nitrogen and sulfur for high quantum yield and excitation-independent emission Angewandte Chemie International Edition 52 7800 7804 23761198 10.1002/anie.201301114

[ref29] Choi Y Kang B Lee J Kim S Kim GT 2016 Integrative approach toward uncovering the origin of photoluminescence in dual heteroatom-doped carbon nanodots Chemistry of Materials 28 6840 6847

[ref30] Lin C Zhuang Y Li W Zhou TL 2019 Blue, green, and red full-color ultralong afterglow in nitrogen-doped carbon dots Nanoscale 11 6584 6590 30601528 10.1039/c8nr09672d

[ref31] Yue YX Wang BL Wang SS Jin CX Lu JY 2020 Boron-doped carbon nanodots dispersed on graphitic carbon as high-performance catalysts for acetylene hydrochlorination 56 5174 5177 10.1039/c9cc09701e 32267259

[ref32] Tian Z Tian P Zhou X Zhou G Mei S 2019 Ultraviolet-pumped white light emissive carbon dot based phosphors for light-emitting devices and visible light communication Nanoscale 11 3489 3494 30742186 10.1039/c9nr00224c

[ref33] Qu S Wang X Lu Q Liu X Wang L 2012 A biocompatible fluorescent ink based on water-soluble luminescent carbon nanodots Angewandte Chemie International Edition 51 12215 12218 23109224 10.1002/anie.201206791

[ref34] Wang F Chen P Feng Y Xie Z Liu Y 2017 Facile synthesis of N-doped carbon dots/g-C3N4 photocatalyst with enhanced visible-light photocatalytic activity for the degradation of indomethacin Applied Catalysis B: Environmental 207 103 113

[ref35] Zhu S Meng Q Wang L Zhang J Song Y 2013 Highly photoluminescent carbon dots for multicolor patterning, sensors, and bioimaging Angewandte Chemie International Edition 52 3953 3957 23450679 10.1002/anie.201300519

[ref36] Fang S Xia Y Lv K Li Q Sun J 2015 Effect of carbon-dots modification on the structure and photocatalytic activity of g-C3N4 Applied Catalysis B: Environmental 185 225 232

[ref37] Gao Z Lin Z Chen X Zhang H Huang Z 2016 A fluorescent probe based on N-doped carbon dots for highly sensitive detection of Hg2+ in aqueous solutions Analytical Methods 8 2297 2304

[ref38] Qian H Han H Zhang F Guo B Yue Y 2004 Non-catalytic CVD preparation of carbon spheres with a specific size Carbon 42 761 766

[ref39] Zhang H Li W Jin Y Sheng W Hu M 2016 Ru-Co(III)-Cu(II)/SAC catalyst for acetylene hydrochlorination Applied Catalysis B: Environmental 189 56 64

[ref40] Moulder JF Stickle WF Sobol PE Bomben KE 1995 Photochemical reduction of carbonate to formaldehyde on TiO2 powder Chemical Physics Letters 220 7 10

[ref41] Renard L Babot O Saadaoui H 2012 Nanoscaled tin dioxide films processed from organotin-based hybrid materials: an organometallic route toward metal oxide gas sensors Nanoscale 4 6806 6813 23011110 10.1039/c2nr31883k

[ref42] Larciprete R Borsella E De Padova P Faglia G Sberveglieri G 1998 Organotin films deposited by laser-induced CVD as active layers in chemical gas sensors Thin Solid Films 323 291 295

[ref43] De Padova P Fanfoni M Larciprete R Mangiantini M Priori S 1994 A synchrotron radiation photoemission study of the oxidation of tin Surface Science 313 379 391

[ref44] Lewin E Patscheider J 2016 Structure and properties of sputter-deposited Al-Sn-N thin films Journal of Alloys and Compounds 682 42 51

[ref45] Inoue Y Nomiya M Takai O Inoue Y Nomiya M 1998 Physical properties of reactive sputtered tin-nitride thin films Vacuum 51 673 676

[ref46] Vinke P Eijk MVD Verbree M Voskamp AF van Bekkum H. 1994 Modification of the surfaces of a gas-activated carbon and a chemically activated carbon with nitric acid, hypochlorite, and ammonia Carbon 32 675 686

[ref47] Dai B Chen K Wang Y Kang LH Zhu MY 2015 Boron and nitrogen doping in graphene for the catalysis of acetylene hydrochlorination ACS Catslysis 5 2541 2547

[ref48] Shen Z Zhao H Liu Y Kan ZY Xing P 2018 Mercury-free nitrogen-doped activated carbon catalyst: an efficient catalyst for the catalytic coupling reaction of acetylene and ethylene dichloride to synthesize the vinyl chloride monomer Reaction Chemistry & Engineering 3 34 40

[ref49] Nkosi B Adams MD Coville NJ Hutchings GJ 1991 Hydrochlorination of acetylene using carbon-supported gold catalysts: a study of catalyst reactivation Journal of Catalysis 128 378 386

[ref50] Bremer H Lieske H 1985 Kinetics of the hydrochlorination of acetylene on HgCl2/active carbon catalysts Applied Catalysis 18 191 191

[ref51] Qi H Li Q Mo ZS Zhang XT Song LJ MCl2 (M=Hg 2017 catalysed hydrochlorination of acetylene-a density functional theory study Molecular Simulation 43 28 33

[ref52] Kowalewska E 1986 Thermochemical properties of H2SnCl6 complexes. Part I. Thermal behaviour of primary n-alkylammonium hexachlorostannates Thermochimica Acta 101 271 289

[ref53] Andrews L Johnson GL Kelsall BJ 1982 Fourier transform infrared matrix infrared spectra of the C2H2-HX and C2HX-HX hydrogen-bonded complex The Journal of Physical Chemistry 86 3374 3380

[ref54] Huber S Knözinger H 1999 Adsorption of CH-acids on magnesia: An FTIR-spectroscopic study Journal of Molecular Catalysis A: Chemical 141 117 127

[ref55] Hu J Yang Q Yang L Zhang Z Su B 2015 Confining noble metal (Pd, Au, Pt) nanoparticles in surfactant ionic liquids: active non-mercury catalysts for hydrochlorination of acetylene ACS Catalysis 5 6724 6731

[ref56] Shang S Zhao W Wang Y Li X Zhang J 2017 Highly efficient Ru@IL/AC to substitute mercuric catalyst for acetylene hydrochlorination ACS Catalysis 7 510 3520

[ref57] Ren Y Wu B Wang F Li H Lv G 2019 Chlorocuprate(i) ionic liquid as an efficient and stable Cu-based catalyst for hydrochlorination of acetylene Catalysis Science & Technology 9 2868 2878

